# C-Reactive protein is an independent surgical indication marker for appendicitis: a retrospective study

**DOI:** 10.1186/1749-7922-4-36

**Published:** 2009-10-31

**Authors:** Shozo Yokoyama, Katsunari Takifuji, Tsukasa Hotta, Kenji Matsuda, Toru Nasu, Mikihito Nakamori, Naoki Hirabayashi, Hiroyuki Kinoshita, Hiroki Yamaue

**Affiliations:** 1 Second Department of Surgery, Wakayama Medical University, School of Medicine, Wakayama, Japan

## Abstract

**Background:**

This study is an attempt to clarify the role of C-reactive protein (CRP) as a surgical indication marker for appendicitis.

**Methods:**

One hundred and fifty patients who underwent appendectomies and had pathologically confirmed appendicitis were reviewed between May 1, 1999 and September 31, 2007. The correlation between preoperative clinical factors and the actual histological severity, and identify surgical indication markers were assessed by univariate and multivariate analyses.

**Results:**

Univariate analysis showed that only the CRP level significantly differ between the surgical treatment necessary group (gangrenous appendicitis) and the possible non-surgical treatment group (catarrhalis and phlegmonous appendicitis). Multivariate analysis indicated only the CRP level to be a surgical indication marker for acute appendicitis. The receiver-operating characteristic (ROC) curve indicated that the cutoff value of CRP for surgical indication of appendicitis is 4.95 mg/dl.

**Conclusion:**

Only the CRP level is consistent with the severity of appendicitis, and considered to be a surgical indication marker for acute appendicitis.

## Background

The treatment of appendicitis has been primarily managed by surgery. However, for those who present with catarrhalis (inflammation within the mucous membrane), or phlegmonous (inflammation in all layers) appendicitis, initial treatment by non-surgical management has been shown to be safe and effective[1,2]. A recent prospective multi-center randomized controlled trial showed that acute non-perforated appendicitis can be treated successfully with antibiotics[3]. The risk of recurrent appendicitis after non-surgical treatment is 5% to 37% [4-6]. Moreover, a routine interval appendectomy after successful non-surgical treatment is not justified and should be abandoned[7]. On the other hand, complicated appendicitis such as gangrenous (necrotic) appendicitis should be treated with emergency surgery[8]. Clinicians must determine the surgical indications after the diagnosis of appendicitis. This study investigated the possibility of a predictive common blood marker for distinguishing surgically indicated gangrenous (necrotic) appendicitis from catarrhalis (within the mucous membrane), or phlegmonous (in all layers) appendicitis.

In clinical practice, the surgical indications for appendicitis are always difficult. In the diagnosis for appendicitis, not for surgical indication, a common blood analysis including white blood cell counts, neutrophil percentage and serum level of CRP has been demonstrated to be important [9-15]. Some reports indicated that appendicitis is unlikely, when the white blood cells count and CRP value are normal [16-18]. However, no report has evaluated the role of CRP for surgical indication of appendicitis. This study investigated whether CRP is a surgical indication marker as well as a diagnostic marker for the decision of an emergency operation for acute appendicitis.

## Methods

Between May 1, 1999, and September 31, 2007, 150 patients, 93 males and 57 females from 4 to 80 years of age, underwent surgical treatment for acute appendicitis in Wakayama Medical University Hospital. All of them had clinical symptoms of acute appendicitis, and underwent an open appendectomy. The appendiceal histological finings confirmed by experienced pathologists identified three groups; the catarrhalis group included 16 patients with proven acute appendicitis within the mucous membrane, the phlegmonous group included 83 patients with proven acute appendicitis in all layers, the gangrenous group included 51 patients with proven acute appendicitis with necrosis. Peripheral venous blood was drawn when the patients presented at the emergency department for white blood cell counts, neutrophil percentage and C-reactive protein level. The duration between the onset of symptoms and presenting to the emergency department was measured. To identify an independent marker for surgical indication of acute appendicitis, these patients were divided into two groups that surgery necessary group for necrotic appendicitis consisted of patients with gangrenous appendicitis and possible non-surgical treatment group for non necrotic appendicitis including catarrhalis and phlegmonous. Univariate and multivariate analyses of the data were carried out using the StatView 5.0 statistical analysis software program. Descriptive statistics for continuous variables such as laboratory parameters were calculated and are reported as the means ± SD. The Mann-Whitney U test was used to detect differences among groups. The logistic regression analysis was carried out for multivariate analysis. All tests were considered to be significant at *P *< 0.05. The optimal cutoff point for the severity of appendicitis was determined using ROC analysis.

## Results

The white blood cell counts and neutrophil percentage did not differ among groups (Table 1). The CRP levels in the catarrhalis, phlegmonous and gangrenous group were 0.23 ± 0.27 mg/dl, 4.09 ± 4.33 mg/dl, and 11.47 ± 7.59 mg/dl, respectively (table 1). The CRP levels were found to be significantly different between the catarrhalis group and the phlegmonous group (0.23 ± 0.27 mg/dl vs. 4.09 ± 4.33 mg/dl, *p *< 0.0001), between the catarrhalis group and the gangrenous group (0.23 ± 0.27 mg/dl vs. 11.47 ± 7.59 mg/dl, *p *< 0.0001), and between the phlegmonous group and the gangrenous group (4.09 ± 4.33 mg/dl vs. 11.47 ± 7.59 mg/dl, *p *< 0.0001). The duration between the onset of symptoms and presentation to the hospital also differed significantly between the catarrhalis group and the phlegmonous group (8.19 ± 5.33 hours vs. 28.27 ± 37.77 hours, *p *< 0.05), between the catarrhalis group and the gangrenous group (8.19 ± 5.33 hours vs. 34.39 ± 27.42 hours, *p *< 0.0001), between the phlegmonous group and the gangrenous group (28.27 ± 37.77 hours vs. 34.39 ± 27.42 hours, *p *< 0.05).

**Table 1:** Comparison Between the Actual Histological Severities and Laboratory Findings

	**Actual Pathologic Diagnosis**		
	
	**Catarrhalis** **(n = 16)**	**Phlegmonous** **(n = 83)**	**Gangrenous** **(n = 51)**
CRP*1 level (mg/dl)	0.23 ± 0.27	4.09 ± 4.33	11.47 ± 7.59
WBC*2 (×100 mm3)	144.69 ± 49.91	139.88 ± 41.87	143.49 ± 47.69
**Neutrophil Percentage (%)**	83.1 ± 7.0	84.4 ± 5.9	86.2 ± 6.5
Duration*3 (hours)	8.19 ± 5.33	28.27 ± 37.77	34.39 ± 27.42

*1 CRP, C-reactive Protein; *2 WBC, White Blood Cell;

*3 Duration, duration between onset of symptoms and hospitalization

**Figure 1:**
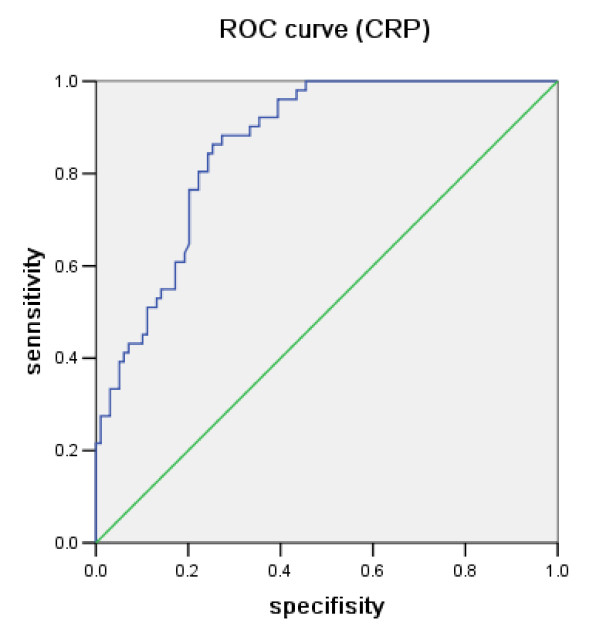
**Receiver-operating characteristic (ROC) curve for serum C-reactive protein (CRP) levels of necrotic appendicitis**.

To elucidate the surgical indication markers for acute appendicitis, the patients were divided into two groups which were surgical treatment necessary group consisted of gangrenous appendicitis and possible non-operative treatment group consisted of catarrhalis and phlegmonous appendicitis, since gangrenous appendicitis cannot be restored to normal histology, and catarrhalis and phlegmonous appendicitis could be curable with antibiotics. The CRP level and duration between the onset of symptoms and hospitalization significantly differed between the surgical treatment necessary and unnecessary group in univariate analysis (table 2). Multivariate analysis of the surgical treatment necessary and unnecessary groups was performed to identify an independent marker for the surgical indications of acute appendicitis. The logistic regression analysis indicated that only the CRP level is an independent marker for distinguishing the severity of acute appendicitis (table 3). The ROC curve showed that the area under the ROC curve for the CRP level of necrotic appendicitis was 0.862, and the optimal cutoff value of CRP for surgical indication for classifying cases was around 4.95 mg/dl (sensitivity = 84.3%, specificity = 75.8%, false positive rate = 24.2%, false negative rate = 15.7%, positive predictive value = 64.2%, negative predictive value = 90.4%; figure 1).

**Table 2:** Comparison Between the Necrotic and Non-necrotic Appendicitis groups by Univariate analysis

	**without necrosis**	**with necrosis**	**P value**
	**(catarrhalis+phregmonous, n = 99)**	**(gangrenous, n = 51)**	
CRP*1 level (mg/dl)	3.462 ± 4.208	11.472 ± 7.594	< 0.0001
WBC*2 (×100 mm3)	140.66 ± 43.03	143.49 ± 47.68	0.713
**Neutrophil Percentage (%)**	84.2 ± 6.0	86.2 ± 6.5	0.1169
Duration*3 (hours)	25.02 ± 35.40	34.40 ± 27.42	0.1007

*1 CRP, C-reactive Protein; *2 WBC, White Blood Cell;

*3 Duration, duration between onset of symptoms and hospitalization

**Table 3:** Comparison Between the Necrotic and Non-necrotic Appendicitis groups by Multivariate analysis

	**P value**	RR*^4 ^(95% CI*^5^)
CRP*^1 ^level (mg/dl)	< 0.0001	1.442 (1.242-1.673)
WBC*^2 ^(×100 mm3)	0.1751	0.988 (0.971-1.005)
**Neutrophil Percentage (%)**	0.3563	1.052 (0.945-1.171)
Duration*^3 ^(hours)	0.3019	0.990 (0.970-1.009)
**Age (<16)**	0.5205	1.507 (0.431-5.261)
**Gender (female)**	0.1799	2.282 (0.683-7.617)

*1 CRP, C-reactive Protein; *2 WBC, White Blood Cell; *3 Duration, duration

between onset of symptoms and hospitalization; *4 RR, Relative risk;

*5 CI, Confidence interval

## Discussion

Appendicitis has been mainly treated by surgical management. However, non-surgical treatment of appendicitis has also been documented with good success[19,20]. The traditional practice of an interval appendectomy has been called into question by some, indicating that patients who do not have recurrent episodes of appendicitis within 3 to 6 months may never need an appendectomy[20]. Therefore, the clinician often wonders whether a patient with appendicitis needs to receive surgical treatment or to be managed with antibiotics. After a patient is diagnosed with appendicitis, clinician generally want to determine the severity before they can select the optimal treatment. If a clinician could predict the severity of appendicitis, one could determine the therapeutic method and the timing of the operation. A surgical indication marker such as the white blood cell count, neutrophil percentage or CRP would be useful for deciding between treating the patient with surgery or antibiotics. The aim of this study was to evaluate whether blood inflammatory markers predict the severity of appendicitis and to identify an independent marker for the surgical indication of acute appendicitis confirmed with clinical symptoms and other modalities. The current study showed that the white blood cell counts and neutrophil percentage are not useful for surgical indication, whereas univariate analysis indicated that only CRP was significantly different between the surgery necessary group and unnecessary group, and multivariate analysis showed that only CRP was an independent marker for necrotic appendicitis. The ROC curve indicated that the optimal cutoff value of CRP for surgical indication for classifying cases was around 5 mg/dl. These data suggested that clinicians should consider the CRP level when selecting the treatment after the diagnosis of appendicitis.

Our novel findings give additional information for surgical indication for appendicitis. Numerous previous studies have shown that the CRP level enhances the precision of diagnosis of acute appendicitis, but not surgical indication. A large retrospective study has documented that the sensitivity of CRP in these patients is greater than 90%[21]. Furthermore, the negative appendectomy rate is reduced by approximately 8% if surgery is cancels in patients with CRP levels and white blood cell counts within the reference range[22]. Another prospective study[11] has shown that it is important to measure serial CRP levels and white blood cell counts in patients with suspected appendicitis. The sensitivity of CRP levels in predicting appendicitis was 60% on admission and increased to 100% by the fourth blood specimen. Conversely, white blood cell counts exhibited a sensitivity of 95% on admission, but dropped to 75% by the fourth specimen. Other studies[16,23] confirm that an elevated CRP serves as a systemic marker of focal inflammation and infection. In this background, CRP and white blood cell counts are important for the diagnosis for appendicitis. After the diagnosis of appendicitis, the clinician must decide surgery or antibiotics. The current study clearly suggested that CRP leads to precise prediction of the severity of acute appendicitis for treatment. However, CRP is not specific for appendicitis, and one should consider the presence of other diseases such as a diverticulum, inflammation of the ileum, or urogenital and gynecological disorders. Therefore, before using our system for surgical indication, clinicians interpreting clinical information must depend on their subjective experience and modalities such as computed tomography and ultrasonography to establish a diagnosis of appendicitis, and must exclude other causes of symptoms. The cut off level at around 5 mg/dl needs to be handled carefully and may need much higher patient numbers to reach the confident level. If clinical symptoms and image examinations indicate that a patient has appendicitis, a patient with a high CRP level should undergo surgery immediately. And, if the CRP level is negative, then a patient could be managed by non-surgical treatment.

## Conclusion

The CRP level, which is a commonly used clinical tool, has been clearly demonstrated to contribute to the prediction of the severity of appendicitis. Once clinical symptoms and examinations have indicated acute appendicitis, the next important step is decision on the most advantageous treatment. The CRP level, neither the white blood cell counts nor neutrophil percentage, is considered to lead to an appropriate decision on whether surgery or non-surgical treatment.

## Competing interests

The authors declare that they have no competing interests.

## Authors' contributions

SY participated in the design of the study, performed statistical analysis and drafted the manuscript. KT participated in its design and coordination. TH helped to draft the manuscript. KM helped to draft the manuscript. TN helped in the revision of the article. MN performed the surgery. NH performed the surgery. HK performed the surgery. HY helped in the revision of the article, and gave approval for the final write up. All authors read and approved the final manuscript.

## References

[B1] Eriksson S, Granstrom L (1995). Randomized controlled trial of appendicectomy versus antibiotic therapy for acute appendicitis. Br J Surg.

[B2] Oliak D, Yamini D, Udani VM, Lewis RJ, Arnell T, Vargas H, Stamos MJ (2001). Initial nonoperative management for periappendiceal abscess. Dis Colon Rectum.

[B3] Styrud J, Eriksson S, Nilsson I, Ahlberg G, Haapaniemi S, Neovius G, Rex L, Badume I, Granstrom L (2006). Appendectomy versus antibiotic treatment in acute appendicitis. a prospective multicenter randomized controlled trial. World J Surg.

[B4] Brown CV, Abrishami M, Muller M, Velmahos GC (2003). Appendiceal abscess: immediate operation or percutaneous drainage?. Am Surg.

[B5] Yamini D, Vargas H, Bongard F, Klein S, Stamos MJ (1998). Perforated appendicitis: is it truly a surgical urgency?. Am Surg.

[B6] Friedell ML, Perez-Izquierdo M (2000). Is there a role for interval appendectomy in the management of acute appendicitis?. Am Surg.

[B7] Kaminski A, Liu IL, Applebaum H, Lee SL, Haigh PI (2005). Routine interval appendectomy is not justified after initial nonoperative treatment of acute appendicitis. Arch Surg.

[B8] Mason RJ (2008). Surgery for appendicitis: is it necessary?. Surg Infect (Larchmt).

[B9] Thimsen DA, Tong GK, Gruenberg JC (1989). Prospective evaluation of C-reactive protein in patients suspected to have acute appendicitis. Am Surg.

[B10] Dueholm S, Bagi P, Bud M (1989). Laboratory aid in the diagnosis of acute appendicitis. A blinded, prospective trial concerning diagnostic value of leukocyte count, neutrophil differential count, and C-reactive protein. Dis Colon Rectum.

[B11] Eriksson S, Granstrom L, Carlstrom A (1994). The diagnostic value of repetitive preoperative analyses of C-reactive protein and total leucocyte count in patients with suspected acute appendicitis. Scand J Gastroenterol.

[B12] Albu E, Miller BM, Choi Y, Lakhanpal S, Murthy RN, Gerst PH (1994). Diagnostic value of C-reactive protein in acute appendicitis. Dis Colon Rectum.

[B13] Gurleyik E, Gurleyik G, Unalmiser S (1995). Accuracy of serum C-reactive protein measurements in diagnosis of acute appendicitis compared with surgeon's clinical impression. Dis Colon Rectum.

[B14] Korner H, Soreide JA, Sondenaa K (1999). Diagnostic accuracy of inflammatory markers in patients operated on for suspected acute appendicitis: a receiver operating characteristic curve analysis. Eur J Surg.

[B15] Yildirim O, Solak C, Kocer B, Unal B, Karabeyoglu M, Bozkurt B, Aksaray S, Cengiz O (2006). The role of serum inflammatory markers in acute appendicitis and their success in preventing negative laparotomy. J Invest Surg.

[B16] Gronroos JM, Gronroos P (1999). Leucocyte count and C-reactive protein in the diagnosis of acute appendicitis. Br J Surg.

[B17] Yang HR, Wang YC, Chung PK, Chen WK, Jeng LB, Chen RJ (2005). Role of leukocyte count, neutrophil percentage, and C-reactive protein in the diagnosis of acute appendicitis in the elderly. Am Surg.

[B18] Yang HR, Wang YC, Chung PK, Chen WK, Jeng LB, Chen RJ (2006). Laboratory tests in patients with acute appendicitis. ANZ J Surg.

[B19] Bagi P, Dueholm S (1987). Nonoperative management of the ultrasonically evaluated appendiceal mass. Surgery.

[B20] Oliak D, Yamini D, Udani VM, Lewis RJ, Vargas H, Arnell T, Stamos MJ (2000). Nonoperative management of perforated appendicitis without periappendiceal mass. Am J Surg.

[B21] Paajanen H, Mansikka A, Laato M, Kettunen J, Kostiainen S (1997). Are serum inflammatory markers age dependent in acute appendicitis?. J Am Coll Surg.

[B22] Eriksson S, Granstrom L, Bark S (1989). Laboratory tests in patients with suspected acute appendicitis. Acta Chir Scand.

[B23] Andersson RE, Hugander AP, Ghazi SH, Ravn H, Offenbartl SK, Nystrom PO, Olaison GP (1999). Diagnostic value of disease history, clinical presentation, and inflammatory parameters of appendicitis. World J Surg.

